# Income, inflammation and cancer mortality: a study of U.S. National Health and Nutrition Examination Survey mortality follow-up cohorts

**DOI:** 10.1186/s12889-020-09923-8

**Published:** 2020-11-26

**Authors:** Joshua E. Chan, Amandeep K. Mann, Daniel S. Kapp, David H. Rehkopf

**Affiliations:** 1 Division of Primary Care and Population Health, Department of Medicine, Stanford University School of Medicine, 1701 Page Mill Road, Room 229, Palo Alto, Stanford, CA 94304 USA; 2 Division of Gynecologic Oncology, Palo Alto Medical Foundation, Sutter Research Institute, Palo Alto, CA USA; 3 Department of Radiation Oncology, Stanford University School of Medicine, Stanford, CA USA

**Keywords:** Inflammation, C-reactive protein, Fibrinogen, Socioeconomic status, Cancer mortality

## Abstract

**Background:**

To estimate the relationship between inflammatory biomarkers and cancer mortality in a nationally representative sample of the U.S. population while controlling for education, occupation, and income.

**Methods:**

Data were obtained from the U.S. National Health and Nutrition Examination Survey from 1988 to 1994 (*N* = 7817) and 1999–2002 (*N* = 2344). We fit Cox proportional hazard models to examine the relationship between C-reactive protein (CRP) and fibrinogen with cancer mortality.

**Results:**

In the full Cox multivariate model, clinically raised CRP was associated with cancer mortality in NHANES 1988–1994 (> 0.99 mg/dL: 95%CI: 1.04–2.13). However, across two inflammatory biomarkers (CRP and Fibrinogen), two NHANES time periods (1998–1994 and 1999–2002) and three income levels (12 strata in total), Hazard ratio confidence intervals did not include the null only for one association: CRP and cancer mortality among low income participants from 1988 to 1994 (HR = 1.83, 95% CI: 1.10–3.04).

**Conclusions:**

We find evidence that only in one unique stratum is earlier life CRP, and not fibrinogen, associated with prospective cancer mortality. After more complete control for socioeconomic confounding, CRP and fibrinogen do not predict cancer mortality in most subpopulations.

**Supplementary Information:**

The online version contains supplementary material available at 10.1186/s12889-020-09923-8.

## Background

Prior studies have found a relationship between higher levels of inflammation and worse cancer prognosis [1, 2]. C-reactive protein (CRP) and fibrinogen are acute-phase proteins that are used to represent low-grade inflammation and associated with cancer incidence and mortality [3–5]. CRP has been correlated with an accumulation of myeloid derived cells with suppressor functions, which are linked to several pathologies, and a heightened risk of cancer mortality, particularly in colorectal cancer [6, 7]. Furthermore, systemic inflammation, especially marked by high CRP levels, has been associated with higher morbidity and mortality among older ages [8, 9]. Fibrinogen is another inflammatory marker that is associated with increased risk and poorer prognosis in epithelial ovarian and other cancers [10].

However, the impact of socioeconomic position (SEP) on these relationships is unclear. Socioeconomic position could act as a confounder or effect modifier of these relationships. One study found a 25% increase in CRP levels among participants who belonged to disadvantaged families during childhood [11]. In addition, Rachet et al. and Coleman et al. have found lower socioeconomic status to be associated with higher cancer mortality [12, 13]. Population-based studies have suggested a correlation between social behaviors and inflammation as risk factors for cancer and other chronic diseases [14]. Lower SEP and certain socio-behavioral patterns have been shown to be inversely correlated with CRP and fibrinogen, and higher systemic inflammation increases the risk of cancer diagnosis [14, 15]. Another report also found a relationship between lower SEP and worse cancer prognosis in melanoma patients [16]. The authors identified several confounders associated with melanoma survival; confounding variables included SEP, education level, and gender, all of which we measured in our study. However, there is limited information on the impact of inflammation on cancer prognosis adjusted for multiple measures of SEP. Previous publications lacked information on occupation, which has previously been evaluated as a predictor for socioeconomic status and life-space mobility [17–19]. Furthermore, previous reports lacked detailed information on demographics including race, body mass index, physical activity, and behavioral factors that could also act as confounders. For example, non-Hispanic blacks and Hispanic subgroups have been shown to have higher inflammatory marker levels compared to non-Hispanic whites [20]. Another study showed physical activity to be associated with improved survival across multiple cancers [21].

Given the limitations of these prior reports, we proposed to investigate whether 1) there is a meaningful relationship between inflammatory biomarkers and cancer mortality after controlling for multiple measures of SEP, and 2) whether family income acts as an effect modifier of the relationships between CRP and fibrinogen with cancer mortality. Finally, although our cohorts varied with respect to sample size, we proposed to determine whether temporal trends existed in the relationships between inflammatory biomarkers, socioeconomic status, and cancer mortality across two time periods.

## Methods

### Data source

Our study’s population-based data are from the U.S. National Health and Nutrition Examination Survey (NHANES) from 1988 to 1994 (NHANES III) and 1999–2002 [22]. Conducted by the Centers for Disease Control and Prevention (CDC), NHANES examines the health and nutrition of individuals living in the U.S. by incorporating a multistage stratified, clustered probability sample of the U.S. civilian population. Data are collected through household interviews and in a mobile examination center (MEC). We use publicly available NHANES data linked to the National Death Index (NDI) to assess cause-specific mortality.

### Inclusion/exclusion criteria

From the full sample of NHANES III (*N* = 33,994) and NHANES 1999–2002 (*N* = 21,004), our data were limited due to NHANES collecting fibrinogen levels of participants aged 40 and over. After including participants from NHANES III (*N* = 8932) in this age group, we excluded those with prior history of cancer (*N* = 1045) and missing information on cancer history (*N* = 3). Participants with CRP ≥ 10 mg/dL and above were also excluded (*N* = 8) because abnormally high CRP levels may be related to active infection rather than chronic inflammation [23]. Due to NHANES reporting all participants who are ≥90 years old as “90,” the participants listed as 90 years old and above were excluded (*N* = 86). The final NHANES III sample consisted of 7817 participants. Of the eligible participants in NHANES 1999–2002 (*N* = 2818), we excluded participants who reported pregnancy (*N* = 45), prior history of cancer (*N* = 334), had a missing history of cancer (*N* = 1), and had CRP levels 10 mg/dL and above (*N* = 4). In addition, we excluded participants ≥85 years old who were reported as “85” in the NHANES 1999–2002 database (*N* = 124). The final NHANES 1999–2002 analytic sample was 2344 participants.

### Demographic and behavioral characteristics

Participants were grouped as non-Hispanic white, non-Hispanic black, or another race (includes Mexican/Hispanic, mixed race, and other race). Body mass index (BMI) was categorized as: underweight (< 18.5 kg/m^2^), normal (18.5–24.9 kg/m^2^), overweight (25.0–29.9 kg/m^2^), and obese (≥30.0 kg/m^2^); physical activity as: lower, about the same, or higher compared to those of similar age based on physical activity questionnaire (PAQ); smoking as: current, former, and never.

### SEP indicators

Participants’ level of education was categorized as less than high school, high school, and above high school.

Poverty-income ratio (PIR), the ratio of family income to the poverty threshold, was used to define income. Our income categories are based on the U.S. Census defined income levels as poverty (ratio < 1), low income (1.0 ≤ ratio < 2.0), middle income (2.0 ≤ ratio < 4.0), and high income (ratio ≥ 4.0) [24]. The “poverty” and “low income” groups were merged together in our analysis. We also created a measure of occupation based on the registrar general’s class-based categorization with the categories of “Not Working”, “White Collar and Professional”, “White Collar, Semi-Routine”, “Blue Collar, High Skill”, and “Blue Collar, Semi-Routine” (Supplemental Table 1) [25]. The registrar general included labor force participants, classified as those who worked or were employed in a job or business within the last two weeks and excluding participants who solely performed housework.

### Inflammatory markers

The serum inflammatory biomarker CRP was assessed by forming an antigen-antibody complex with latex particles coated with anti-CRP antibodies. A Behring Nephelometer measured light scattering after six minutes. CRP levels were then calculated using a calibration curve. The inflammatory biomarker fibrinogen levels were measured through thrombin clotting time [22]. In this assay, the enzyme thrombin was used to convert fibrinogen into its insoluble polymer, fibrin. In this study, CRP and fibrinogen levels were used as inflammatory biomarkers to evaluate their effects on cancer prognosis across income. The intermediate ranges for CRP are 0.22–0.99 mg/dL and 200–400 mg/dL for fibrinogen [26–28].

In our sample, the mean CRP level was 0.45 mg/dL (0.21–9.79 mg/dL) in NHANES III and 0.45 mg/dL (0.01 to 7.83 mg/dL) in NHANES 1999–2002. The mean fibrinogen level was 303 mg/dL (19–957 mg/dL) in NHANES III and 367 mg/dL (120–808 mg/dL) in NHANES 1999–2002. Due to the majority of the participants having CRP levels below the baseline detection level of 0.22 mg/dL and the NHANES labeling any value below 0.22 mg/dL as 0.21 mg/dL, CRP was classified as a categorical variable. Using Swede et al. and Visser et al.’s classification methods, we categorized CRP levels of < 0.22 mg/dL, 0.22–0.99 mg/dL, and ≥ 1.00 mg/dL as undetected, intermediate, and clinically raised CRP, respectively [26, 28]. Fibrinogen level was treated as a continuous variable in the test of association and multivariate analysis due to the normal distribution of participants’ fibrinogen levels. However, for the survival curves, the intermediate range of fibrinogen was used as the cutoff [27].

### Measurement for mortality endpoint

We obtained data on causes, month, and year of death from the National Center for Health Statistics (NCHS) linkage to the NDI, with follow-up through December 31, 2015 [22]. These data were linked to the NHANES dataset through probabilistic matching with social security number, birth date, and other personal data. The International Classification of Diseases codes for death were used to determine underlying causes of death for all cancer sites (C00-C97).

### Statistical analysis

Strata, cluster, and weight variables were used to ensure that oversampling of any groups did not occur. For the NHANES III dataset, we used SDPSTRA6, SDPPSU6, and WTPFHX6 as strata, cluster, and weight variables, respectively. Similarly, with the NHANES 1999–2002 dataset, SDMVSTRA, SDMVPSU, and WTMEC4YR were used as strata, cluster, and weight variables for analysis use. Using the appropriate Chi-square and t-tests, the associations with income levels were examined. Additionally, we analyzed the associations of demographic, socioeconomic, and behavioral characteristics in our study with CRP and fibrinogen levels (Supplemental Table 2) (Supplemental Table 3). We created unadjusted Kaplan-Meier curves to investigate the differences in cancer-specific survival in the overall income level groups and inflammation. Furthermore, income level groups were stratified to examine how the difference in survival of participants varied by CRP and fibrinogen level. Age was used as a baseline for time to follow-up, death from other causes or cancer death [29]. Additionally, we utilized Cox-proportional hazard models to adjust for potential confounders of CRP and fibrinogen levels for cancer mortality. Due to the strong correlation between CRP and fibrinogen (NHANES III: B = 0.003, *p* < 0.001; NHANES 1999–2002: B = 0.004, *p* < 0.001) and the uncertain nature of their causal ordering, separate multivariable analyses were performed for these inflammatory markers. Cox-proportional hazard models were fit to test for cancer mortality associations for each variable, represented as Model 1 in Supplemental Table 4. Furthermore, 3 models were fit based on a priori specification for demographic (race and gender), socioeconomic (income, education, and occupation), and behavioral (BMI, physical activity, and smoking status) factors. A Directed Acyclic Graph (DAG) was constructed to describe the possible confounders in the association between the inflammatory biomarkers and cancer mortality (Supplemental Fig. 1).

Proportional hazard assumptions were assessed for all covariates in the full model. Since NHANES III and NHANES 1999–2002 were separately grouped by strata, cluster, and weights, the two datasets were analyzed separately. We examined the association of CRP and fibrinogen levels with cancer mortality in the overall multivariate analysis. In the subset analyses, the Cox-proportional hazard model was stratified by income levels and gender to investigate the association between inflammation and cancer mortality in each income group. Data analyses were conducted using SAS® 9.3 (SAS Institute Inc., Cary, NC, USA). This study was exempted from the IRB approval because it is a public use data file and does not contain identifying information of participants.

## Results

Of 7817 participants (median age: 53 years; range: 40–89) in the NHANES III sample, 79.0% were white, 9.8% were black, and 11.1% were of another race. The sample consists of 52.4% females and 47.6% males. About 61.6% of participants had education levels of high school or below, while 38.4% of participants had education levels above high school. Low, middle, and high-income levels were represented in 43.8, 38.6, and 17.7% of participants, respectively (Table 1). The mean follow-up time for low, middle and high-income levels was 7.9 years, 7.8 years, and 7.4 years ranging between 3.3 and 9 years.

**Table 1:** Demographics, Socioeconomic Status, and Behavioral Characteristics by Income Levels from NHANES III (1988–1994)

Characteristics	Total (***N*** = 7817)	Low Income (***N*** = 3420)	Middle Income (***N*** = 3014)	High Income (***N*** = 1383)	***P***-value
***Age (years)***					< 0.001^a^
Median (Range)	53 (40–89)	58 (40–89)	53 (40–89)	51 (40–89)	
Younger than 55 yrs	51.6%	40.7%	52.0%	61.0%	
55 yrs. or older	48.4%	59.3%	48.0%	39.0%	
***Race/Ethnicity***					< 0.001^a^
White	79.0%	64.1%	80.5%	90.5%	
Black	9.8%	17.7%	8.7%	4.2%	
Hispanics/Other	11.1%	18.2%	10.7%	5.3%	
***Gender***					< 0.001^a^
Male	47.6%	42.3%	47.3%	52.8%	
Female	52.4%	57.7%	52.7%	47.2%	
***Education***					< 0.001^a^
Below High School	29.1%	55.6%	29.6%	14.8%	
High School/Equivalent	32.5%	27.1%	36.6%	26.2%	
Above High School	38.4%	7.9%	29.2%	62.9%	
***Occupation***					< 0.001^a^
Not Working	2.8%	5.3%	2.4%	1.1%	
White Collar and Professional	26.7%	11.7%	23.5%	45.0%	
White Collar, Semi-Routine	22.8%	15.6%	23.7%	27.9%	
Blue Collar, High Skill	13.3%	14.3%	15.1%	9.6%	
Blue Collar, Semi-Routine	34.5%	53.1%	35.3%	16.4%	
***Body Mass Index (kg/m***^***2***^***)***					0.001^a^
Median (Range)	26.6 (11.7–67.3)	27.3 (13.3–60.0)	26.5 (11.7–67.3)	25.9 (15.9–55.9)	
Underweight	1.6%	2.2%	1.5%	1.0%	
Normal	34.5%	31.3%	35.0%	36.6%	
Overweight	37.1%	34.3%	37.2%	39.5%	
Obese	26.9%	32.2%	26.4%	22.8%	
***Smoking***					< 0.001^a^
Never	42.2%	41.8%	43.2%	41.0%	
Former	33.9%	28.9%	33.4%	39.3%	
Current	23.9%	29.4%	23.4%	19.7%	
***Physical Activity***^***b***^					< 0.001^a^
More Active	37.7%	28.4%	37.9%	45.8%	
Less Active	21.6%	27.8%	20.5%	17.5%	
About Same	40.7%	43.8%	41.6%	36.7%	
***C-Reactive Protein***					< 0.001^a^
Mean (Range)	0.45 (0.21–9.79)	0.57 (0.21–9.79)	0.43 (0.21–8.90)	0.38 (0.21–9.20)	
< 0.22 mg/dL	66.1%	56.0%	67.9%	72.8%	
0.22–0.99 mg/dL	24.7%	30.7%	23.7%	20.7%	
> 0.99 mg/dL	9.2%	13.3%	8.4%	6.5%	
***Fibrinogen***					< 0.001^c^
Mean (Range)	303 (19–957)	319 (30–928)	298 (19–957)	294 (85–806)	

^a^ Chi-Square tests were performed for categorical variables

^b^ Physical activity compared to others of the same age

^c^ t-tests were performed for the continuous variables

Of 2344 participants (median age: 52 years; range: 40–84) in the NHANES 1999–2002 sample, 74.5% were white, 10.6% were black, and 14.8% were of another race. 51.6% of participants were female, and 48.4% male. Roughly 49.5% of participants had education levels of high school or below, whereas 50.4% of participants were educated above high school. Low, middle, and high-income levels were represented in 38.6, 35.4, and 26.0% of participants, respectively (Table 2). The mean follow-up time for low, middle and high-income levels was 7.7 years, 7.4 years, and 7.3 years ranging between 3.3 and 8.3 years.

**Table 2:** Demographics, Socioeconomic Status, and Behavioral Characteristics by Income Levels from NHANES 1999–2002

Characteristics	Total (***N*** = 2344)	Low Income (***N*** = 904)	Middle Income (***N*** = 830)	High Income (***N*** = 610)	***P***-value
***Age (years)***					< 0.001^a^
Median (Range)	52 (40–84)	55 (40–84)	53 (40–84)	51 (40–84)	
Younger than 55 yrs	55.5%	48.5%	51.7%	64.0%	
55 yrs. or older	44.5%	51.5%	48.3%	36.0%	
***Race/Ethnicity***					< 0.001^a^
White	74.5%	60.5%	71.7%	87.3%	
Black	10.6%	14.9%	12.5%	5.9%	
Hispanics/Other	14.8%	24.6%	15.8%	6.8%	
***Gender***					0.03^a^
Male	48.4%	43.7%	50.1%	50.4%	
Female	51.6%	56.3%	49.9%	49.6%	
***Education***					< 0.001^a^
Below High School	24.3%	46.2%	24.3%	8.5%	
High School/Equivalent	25.2%	27.8%	28.0%	20.8%	
Above High School	50.4%	26.0%	47.7%	70.7%	
***Occupation***					< 0.001^a^
Not Working	2.1%	4.8%	2.2%	0.2%	
White Collar and Professional	32.1%	17.1%	25.4%	49.1%	
White Collar, Semi-Routine	23.6%	17.4%	24.6%	27.2%	
Blue Collar, High Skill	13.4%	15.5%	15.4%	10.0%	
Blue Collar, Semi-Routine	28.8%	45.1%	32.3%	13.5%	
***Body Mass Index (kg/m***^***2***^***)***					0.64^a^
Median (Range)	27.7 (12.0–66.4)	28.1 (15.8–66.4)	27.9 (12.0–63.9)	27.4 (16.5–61.2)	
Underweight	1.1%	1.5%	1.1%	0.9%	
Normal	27.7%	26.8%	26.8%	29.1%	
Overweight	37.4%	34.7%	39.4%	37.5%	
Obese	33.8%	37.0%	32.6%	32.5%	
***Smoking***					0.002^a^
Never	45.5%	44.4%	43.1%	48.5%	
Former	31.7%	26.2%	34.2%	33.5%	
Current	22.8%	29.4%	22.7%	18.0%	
***Physical Activity***^***b***^					0.001^a^
More Active	40.6%	36.0%	44.7%	40.2%	
Less Active	20.8%	27.1%	19.3%	17.7%	
About Same	38.6%	37.0%	36.0%	42.1%	
***C-Reactive Protein***					< 0.001^a^
Mean (Range)	0.45 (0.01–7.83)	0.52 (0.01–7.83)	0.46 (0.01–6.90)	0.39 (0.01–6.80)	
< 0.22 mg/dL	44.9%	38.9%	43.7%	50.5%	
0.22–0.99 mg/dL	44.2%	45.5%	45.3%	42.2%	
> 0.99 mg/dL	10.9%	15.5%	11.1%	7.3%	
***Fibrinogen***					0.003^c^
Mean (Range)	367 (120–808)	385 (173–808)	371 (177–747)	351 (120–715)	

^a^ Chi-Square tests were performed for categorical variables

^b^ Physical activity compared to others of the same age

^c^ t-tests were performed for the continuous variables

In the NHANES III dataset, lower income was associated with older age (*p* < 0.001), black or other non-white participants (*p* < 0.001), and those with education levels of high school or below (*p* < 0.001). (Table 1).

Similarly, for participants in the NHANES 1999–2002 dataset, those in the lower income cohort were older (*p* < 0.001), black or non-white (p < 0.001), and educated at or below the high school level (*p* < 0.001).

We found an inverse relationship between income levels and both CRP and fibrinogen levels in both time periods. For the low, middle, and high income levels in NHANES III, the mean CRP levels were 0.57 mg/dL, 0.43 mg/dL, and 0.38 mg/dL (p < 0.001), and the mean fibrinogen levels were 319 mg/dL, 298 mg/dL, and 294 mg/dL, respectively (p < 0.001) (Table 1). For the low, middle, and high income levels in NHANES 1999–2002, the mean CRP levels were 0.52 mg/dL, 0.46 mg/dL, and 0.39 mg/dL (*p* = 0.003), and the mean fibrinogen levels were 385 mg/dL, 371 mg/dL, and 351 mg/dL, respectively (*p* = 0.003) (Table 2).

We found no statistically significant relationship between income levels and cancer mortality (Supplemental Fig. 2). We examined the relationship between inflammatory markers and cancer mortality and found that those with higher CRP levels had a significantly higher risk of cancer mortality in the NHANES III dataset (log-rank *p* < 0.001), yet there was no significant association between CRP and cancer mortality in the NHANES 1999–2002 dataset (log-rank *p* = 0.10) (Supplemental Fig. 3). Similarly, higher fibrinogen levels were correlated to higher risk of cancer mortality in the NHANES III dataset (log-rank *p* = 0.03), but the correlation was not statistically significant in the NHANES 1999–2002 dataset (log-rank *p* = 0.28) (Supplemental Fig. 4).

In the full Cox multivariate model, clinically raised CRP was associated with cancer mortality in the NHANES III (> 0.99 mg/dL: 95%CI: 1.04–2.13) (Table 3). The relationship between CRP and cancer mortality was not significant in the NHANES 1999–2002 cohort, regardless of socioeconomic status (0.22–0.99 mg/dL: 95%CI: 0.55–1.59) (> 0.99 mg/dL: 95%CI: 0.92–2.42) (Table 3). Fibrinogen was also not associated with cancer mortality in both the NHANES III (95%CI: 1.000–1.002) and NHANES 1999–2002 (95%CI: 0.998–1.002) samples (Table 4).

**Table 3:** Multivariate Cox Proportional Hazard Model Adjusted for CRP

Characteristics	NHANES III 1988–1994			NHANES 1999–2002		
	Hazard Ratio	95% Confidence Interval	P-value	Hazard Ratio	95% Confidence Interval	***P***-value
***C-Reactive Protein (mg/dL)***						
< 0.22 mg/dL^a^	1			1		
0.22–0.99 mg/dL	1.01	0.81–1.26	0.90	0.94	0.55–1.59	0.80
> 0.99 mg/dL	1.49	1.04–2.13	0.03	1.49	0.92–2.42	0.10
***Race/Ethnicity***						
White^a^	1			1		
Black	1.25	0.97–1.61	0.09	1.29	0.75–2.23	0.35
Hispanics/Other	0.90	0.61–1.34	0.61	1.30	0.64–2.64	0.46
***Gender***						
Male^a^	1			1		
Female	0.68	0.54–0.87	0.003	0.52	0.27–0.99	0.047
***Education***						
Below High School^a^	1			1		
High School/Equivalent	1.31	0.99–1.72	0.06	1.19	0.57–2.52	0.63
Above High School	1.17	0.79–1.74	0.41	0.73	0.31–1.68	0.44
***Income Level***						
Low^a^	1			1		
Middle	0.85	0.70–1.05	0.12	1.20	0.70–2.05	0.49
High	0.79	0.56–1.11	0.17	1.58	0.78–3.19	0.20
***Occupation***						
Blue Collar, Semi-Routine^a^	1			1		
White Collar and Professional	0.89	0.60–1.32	0.54	0.73	0.40–1.31	0.28
White Collar, Semi-Routine	0.94	0.60–1.46	0.76	1.04	0.57–1.91	0.89
Blue Collar, High Skill	1.05	0.67–1.62	0.84	1.31	0.88–1.97	0.18
Not Working	1.34	0.78–2.30	0.28	0.91	0.26–3.11	0.87
***Body Mass Index (kg/m***^***2***^***)***						
Underweight^a^	1			1		
Normal	1.40	0.73–2.67	0.31	1.71	0.33–8.86	0.51
Overweight	1.10	0.61–1.99	0.74	1.91	0.41–8.81	0.40
Obese	1.64	0.84–3.17	0.14	2.58	0.52–12.72	0.24
***Smoking***						
Never^a^	1			1		
Former	1.67	1.15–2.42	0.01	2.23	1.32–3.77	0.004
Current	3.86	2.87–5.18	< 0.001	6.99	4.11–11.88	< 0.001
***Physical Activity***^***b***^						
About the Same^a^	1			1		
Less Active	1.15	0.86–1.53	0.35	1.15	0.57–2.32	0.68
More Active	0.69	0.55–0.85	0.001	0.87	0.47–1.62	0.65

^a^ Reference group

^b^ Physical activity compared to others of same age

**Table 4:** Multivariate Cox Proportional Hazard Model Adjusted for Fibrinogen

Characteristics	NHANES III 1988–1994			NHANES 1999–2002		
	Hazard Ratio	95% Confidence Interval	***P***-value	Hazard Ratio	95% Confidence Interval	***P***-value
***Fibrinogen (mg/dL)***	1.001	1.000–1.002	0.07	1.00	0.998–1.002	0.75
***Race/Ethnicity***						
White^a^	1			1		
Black	1.26	0.98–1.63	0.07	1.30	0.74–2.27	0.35
Hispanics/Other	0.89	0.60–1.32	0.56	1.27	0.62–2.58	0.50
***Gender***						
Male^a^	1			1		
Female	0.70	0.54–0.89	0.005	0.52	0.27–1.01	0.05
***Education***						
Below High School^a^	1			1		
High School/Equivalent	1.31	1.00–1.73	0.05	1.18	0.57–2.46	0.65
Above High School	1.18	0.80–1.75	0.40	0.70	0.31–1.60	0.39
***Income Level***						
Low^a^	1			1		
Middle	0.85	0.70–1.04	0.11	1.19	0.69–2.04	0.53
High	0.78	0.55–1.11	0.16	1.52	0.76–3.04	0.23
***Occupation***						
Blue Collar, Semi-Routine^a^	1			1		
White Collar and Professional	0.89	0.59–1.32	0.55	0.75	0.43–1.31	0.30
White Collar, Semi-Routine	0.93	0.60–1.45	0.75	1.10	0.62–1.94	0.73
Blue Collar, High Skill	1.03	0.67–1.59	0.89	1.30	0.87–1.95	0.20
Not Working	1.31	0.76–2.25	0.32	0.94	0.28–3.14	0.91
***Body Mass Index (kg/m***^***2***^***)***						
Underweight^a^	1			1		
Normal	1.36	0.71–2.59	0.35	1.84	0.37–9.25	0.44
Overweight	1.08	0.60–1.94	0.80	2.07	0.45–9.51	0.34
Obese	1.63	0.86–3.11	0.13	2.84	0.58–14.04	0.19
***Smoking***						
Never^a^	1			1		
Former	1.69	1.16–2.45	0.01	2.24	1.32–3.80	0.004
Current	3.87	2.87–5.23	< 0.001	7.17	4.23–12.16	< 0.001
***Physical Activity***						
About the Same^a^	1			1		
Less Active	1.15	0.85–1.55	0.35	1.19	0.60–2.35	0.61
More Active	0.69	0.55–0.85	0.001	0.90	0.47–1.70	0.73

^a^ Reference group

^b^ Physical activity compared to others of same age

**Fig. 1:**
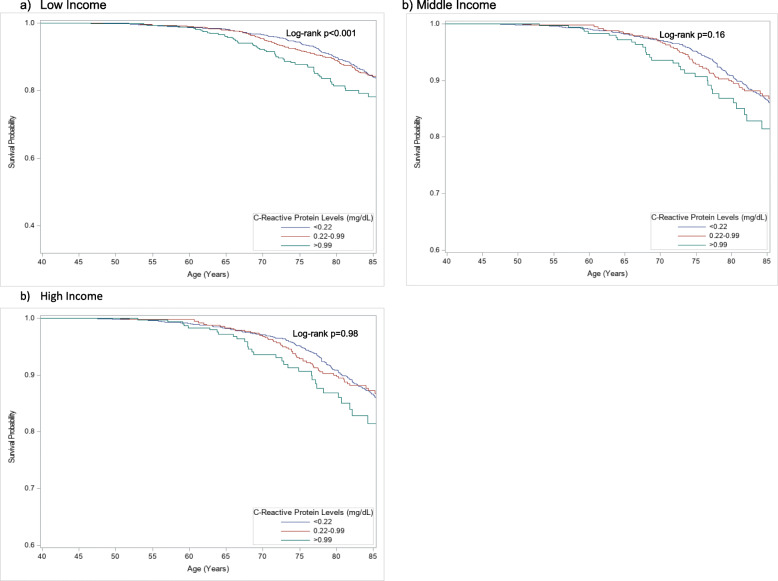
Kaplan-Meier Freedom from Cancer-Specific Mortality Curves of CRP in NHANES III, Stratified by Income Level. Among low-income participants, high CRP was correlated to higher cancer mortality

In the multivariate analysis evaluating CRP, being female was associated with lower cancer mortality compared to being male in both the NHANES III (HR = 0.68, 95%CI: 0.54–0.87; *p* = 0.003) and the NHANES 1999–2002 (HR = 0.52, 95%CI: 0.27–0.99; *p* = 0.047) datasets (Table 3). In addition, in the analysis evaluating fibrinogen, being female was associated with lower cancer mortality in the NHANES III (HR = 0.70, 95%CI: 0.54–0.89; *p* = 0.005) (Table 4). We further stratified the full Cox multivariate model by income groups to assess for associations of each income group with inflammation levels and cancer mortality. After adjusting for all factors, we found that higher CRP levels were associated with lower cancer survival among low income participants in the NHANES III dataset (0.22–0.99 mg/dL: HR = 1.23, 95%CI: 0.88–1.74; *p* = 0.22) (> 0.99 mg/dL: HR = 1.83, 95%CI: 1.10–3.04; *p* = 0.02), but the relationship was not significant in NHANES 1999–2002 (Table 5, Fig. 1, Supplemental Fig. 5).

**Table 5:** Multivariate Analysis – Cancer-specific Mortality Adjusted for Inflammatory Markers and Other Factors, Stratified by Income Level

Factors	NHANES III 1988–1994			NHANES 1999–2002		
	Hazard Ratio	95% Confidence Interval	***P***-value	Hazard Ratio	95% Confidence Interval	***P***-value
**Low Income**						
***C-Reactive Protein***^***a***^						
< 0.22 mg/dL	1			1		
0.22–0.99 mg/dL	1.23	0.88–1.74	0.22	0.77	0.35–1.67	0.49
> 0.99 mg/dL	1.83	1.10–3.04	0.02	1.46	0.87–2.45	0.15
***Fibrinogen***^***a***^	1.001	1.000–1.003	0.10	0.997	0.994–1.000	0.08
**Middle Income**						
***C-Reactive Protein***^***a***^						
< 0.22 mg/dL	1			1		
0.22–0.99 mg/dL	0.87	0.63–1.19	0.37	1.05	0.39–2.82	0.92
> 0.99 mg/dL	1.12	0.64–1.97	0.69	0.61	0.17–2.11	0.42
***Fibrinogen***^***a***^	1.000	0.998–1.002	0.67	1.001	0.998–1.01	0.43
**High Income**						
***C-Reactive Protein***^***a***^						
< 0.22 mg/dL	1			1		
0.22–0.99 mg/dL	0.84	0.44–1.63	0.60	1.07	0.53–2.18	0.84
> 0.99 mg/dL	1.41	0.62–3.19	0.41	2.87	0.88–9.37	0.08
***Fibrinogen***^***a***^	1.000	0.997–1.003	0.94	1.002	0.998–1.01	0.36

^a^ Adjusted for gender, race, education, occupation, body mass index, smoking status, and physical activity

## Discussion

In this study of 10,161 participants without a cancer diagnosis at baseline, we found that lower income was associated with increased biomarkers of inflammation. After controlling for measures of socioeconomic position, we found associations between CRP and cancer mortality in the NHANES III sample, but we found non-significant trends in the NHANES 1999–2002 sample.

Our findings that higher CRP and was associated with higher cancer mortality compared to their respective undetected and intermediate levels, but also that undetected and intermediate inflammation levels were associated with similar cancer survival rates, may indicate that having above-average inflammation levels is more closely associated with higher cancer mortality than their respective below-average levels. In addition, it indicates the greater importance that anti-inflammatory drugs may have in lowering cancer mortality perhaps in subgroups with low income and high C-reactive protein levels. Nevertheless, our findings that this only occurred in 1 out of 12 strata of the population caution that this may not be of relevance to current cohorts.

Prior studies have also shown lower income to be associated with inflammation, specifically with elevated levels of CRP and fibrinogen. Nazmi and colleagues performed a systematic review of 32 studies and found that poverty and non-white race were correlated with higher CRP levels [30]. Others found similar results in a cross-sectional study surveyed in the U.S., with higher inflammation in low income participants adjusted for demographics, health status, and behavioral characteristics [31]. In this current report of over 10,000 participants, we also found that lower income correlated with higher CRP levels. In contrast, Yang et al. found that high SEP was associated with increased levels of inflammation and obesity in African-American males [14]. However, their study was focused on young adults. Our analysis incorporated both CRP and fibrinogen and notably adjusted for age, race, gender, BMI, education, occupation, income, and behavioral factors including physical activity and smoking.

The association between lower income and higher CRP levels, but lack of association with fibrinogen levels, may be explained by CRP levels being a more accurate representation of lifetime inflammation. According to Davillas et al., fibrinogen levels vary by other physiological functions of the body [32]. Due to our efforts to minimize confounding, our adjusted sample may have shown a non-significant association between income and fibrinogen due to controlling for confounders that may vary by fibrinogen levels.

Previous reports showed that inflammation and cancer mortality may depend on gender. One study found a positive correlation between CRP levels and cancer mortality, but only in males [33]. In another study of female participants, there was no such correlation [34]. In this current report of over 10,000 men and women, we found that higher CRP was associated with increased cancer mortality among males. However, this association was not shown in female participants. Goyal and colleagues found a similar association between CRP levels and specifically in colorectal cancer mortality [7]. While we found this relationship for the composite of cancers, further studies are warranted to investigate the varying influence of inflammation on different cancer types adjusted for a full range of demographic and socioeconomic confounders. Our data may suggest that inflammation may have a greater impact on those with lower rather than upper SEP. However, caution is warranted for interpretation of these subgroup tests as we performed 12 pre-specified subgroup analyses. The subgroup associations that we found should be tested in another sample.

In addition to finding a correlation between lower income and higher inflammation, we also found a trend that low income was associated with higher cancer mortality, though not statistically significant. This finding supports the conclusions of numerous studies. In an international systematic review, Manser and Bauerfeind found that low SEP colorectal cancer patients had higher mortality [35]. Bristow’s group studied ovarian cancer patients and revealed that black people and women of lower SEP had higher mortality rates compared to white and wealthier women [36]. Our results indicate that the socioeconomic disparities found by these studies were consistent for the composite of all cancers studied in our cohort. It is possible that lower income patients have higher mortality because of poor health conditions in addition to limited access to health care [37].

Lower income participants may have higher inflammation due to higher BMI, decreased physical activity, smoking, and other life stressors altering their inflammatory response [38, 39]. One possible mechanism for the association between inflammation and cancer mortality is the presence of the cyclooxygenase-2 and nuclear factor kappa-B genes, which directly correlates inflammation and cancer [40]. Another biologic rationale is the presence of tumor necrosis factor-alpha and interleukin-6 induced signaling, which can promote tumor growth [40]. In addition, cancers arising from infected regions of the body due to behavioral factors can cause chronic inflammation, further compromising patients’ outcomes. For example, there is a strong connection between gastrointestinal infection, inflammatory bowel diseases and colorectal cancer [1, 41, 42]. Numerous trials and meta-analyses have explored the use of anti-inflammatory agents, including aspirin, in the prevention of cancer and in the reduction of metastasis in patients with cancer [43]. The benefit of anti-inflammatory drugs appears to be cancer site specific, with strong benefits noted for colon, breast, and ovarian cancers [44–46]. However, our data suggest that it may be more prudent to target the use of the anti-inflammatory therapies on those of lower income and/or with elevated baseline immune biomarkers, adjusted by cancer site [47, 48].

### Strengths and limitations

Our study has limitations that may impact how generalizable these results are to all populations. Our cohort only included adults over 40 years and lacked information on specific cancer type, time of diagnosis, stage of disease, surgical and chemotherapy treatment. Without this data, we were unable to perform specific analyses on different cancer types with varying immune and inflammatory responses. In addition, NHANES 1999–2002 had 2344 participants in its sample compared to 7817 in NHANES III. We thus had substantially less power in the more recent NHANES study. However, the trends in the NHANES 1999–2002 data were similar to the statistically significant trends in NHANES III. Additionally, we do not have detailed information on comorbidities such as autoimmune diseases and other chronic illnesses that may alter the inflammatory response. Our study also did not collect biomarker data at various time points in relation to patient’s diagnosis, treatment, and follow-up. Furthermore, CRP and fibrinogen are non-specific acute-phase proteins and are not completely representative of low-grade inflammation. Factors such as age, diet, and genetics may cause variation in inflammatory marker levels [49]. However, the strengths of our study include that it consists of a nationally representative cohort and is a cross-discipline investigation incorporating social science, immunology, and oncology.

Previous studies that did not control for SEP may be confounded. For example, Swede et al. observed that higher baseline CRP was correlated to higher colorectal cancer mortality; however, their findings were not adjusted for SEP [26]. Similarly, Wulaningsih et al. concluded that higher CRP was associated with a greater risk of dying from cancer after only adjusting for BMI and waist circumference [33]. However, by adjusting for education, occupation, and income, we eliminated some additional residual socioeconomic confounding.

The shorter follow-up period of the more recent NHANES sample also limited our study. The temporal differences in our model may simply be due to unstable findings, or it may be due to temporal differences across the NHANES III and NHANES 1999–2002 datasets. Our results are consistent with there being temporal differences in how CRP and fibrinogen predict outcomes. Sin et al. also found a temporal relationship between systemic inflammation and chronic obstructive pulmonary disease mortality, concluding that with 7 to 8 years of surveillance, baseline CRP levels were associated with higher rates of cardiovascular diseases and cancer-specific death [50]. In addition, Oikawa et al. showed that a temporal difference in CRP levels was associated with increased cancer mortality and heart failure [51]. The associations between inflammation and cancer mortality may have been statistically significant in the NHANES III because they were collected over a seven-year period as opposed to four years in NHANES 1999–2002.

## Conclusions

Our results suggest that lower income is associated with higher levels of CRP and fibrinogen. Once controlling for multiple measures of SEP, we found that elevated inflammation levels independently predicted cancer mortality only in low income participants within the NHANES III dataset. This suggests that there is limited benefit of these inflammatory markers for cancer prediction in most populations, and in the more recent cohort. While inflammatory markers may serve as potential targets for novel drug development [52], they do not appear to be productive for predicting cancer once socioeconomic confounding is more completely accounted for.

## Supplementary Information

**Additional file 1: Supplemental Table 1.** Occupational Classifications in NHANES III and NHANES 1999–2002. Occupational Classification

**Additional file 2: Supplemental Table 2.** Demographic, Socioeconomic, and Behavioral Characteristics Associated with CRP Levels. Association analysis between CRP and factors

**Additional file 3: Supplemental Table 3.** Demographic, Socioeconomic, and Behavioral Characteristics Associated with Fibrinogen Levels. Association analysis between fibrinogen and factors

**Additional file 4: Supplemental Table 4.** Association of Cancer Mortality and Inflammatory Markers of All Participants. Cox-proportional hazard analysis of unadjusted (Model 1), demographic adjusted (Model 2), socioeconomic status adjusted (Model 3), and behavioral factors adjusted (Model 4).

**Additional file 5: Supplemental Figure 1.** Directed Acyclic Graph (DAG) depicting causal assumptions of inflammation and cancer mortality. Directed Acyclic Graph describes the relationship between inflammation and cancer mortality as well as potential confounders that is associated with both inflammation and cancer mortality.

**Additional file 6: Supplemental Figure 2.** Kaplan-Meier Survival from Cancer-Specific Mortality Curves by Income Level. Differences in survival outcomes stratified by income levels

**Additional file 7: Supplemental Figure 3.** Kaplan-Meier Survival from Cancer-Specific Mortality Curves by CRP Level. Differences in survival outcomes stratified by CRP levels

**Additional file 8: Supplemental Figure 4.** Kaplan-Meier Survival from Cancer-Specific Mortality Curves by Fibrinogen Level. Differences in survival outcomes stratified by fibrinogen levels

**Additional file 9: Supplemental Figure 5.** Kaplan-Meier Survival from Cancer-Specific Mortality Curves of CRP in NHANES 1999–2002, Stratified by Income Level. Differences in survival outcomes stratified by CRP and income levels

## Data Availability

The datasets generated and/or analyzed during the current study are publicly available from the Centers for Disease Control and Prevention (https://wwwn.cdc.gov/nchs/nhanes/Default.aspx).

## Abbreviations

CRP: C-reactive protein
SEP: Socioeconomic position
NHANES: National Health and Nutrition Examination Survey
CDC: Centers for Disease Control and Prevention
MEC: Mobile Examination Center
NDI: National Death Index
BMI: Body mass index
PIR: Poverty-income ratio
NCHS: National Center for Health Statistics
DAG: Directed acyclic graph
CI: Confidence interval
HR: Hazard ratio
