# Supplementary material for: Income, inflammation and cancer mortality: a study of U.S. National Health and Nutrition Examination Survey mortality follow-up cohorts
Source: BMC Public Health. 2020 Nov 26;20:1805. doi: 10.1186/s12889-020-09923-8 (PMC7689964; doi:10.1186/s12889-020-09923-8)
Supplement: Supplementary file 1 — Additional file 1: Supplemental Table 1. Occupational Classifications in NHANES III and NHANES 1999–2002. Occupational Classification [file 12889_2020_9923_MOESM1_ESM.docx]

Supplemental Table 1. Occupational Classifications in NHANES III and NHANES 1999-2002^11^

| **Category** | **NHANES III occupational category item description and code** | **NHANES 1999-2002 occupational category item description and code** |
| --- | --- | --- |
| White collar and  professional | Executive, administrators, and managers (1), management related occupations (2), engineers and scientists (3), health diagnosing, assessment and treating occupations (4), teachers (5), writers, artists, entertainers, and athletes (6), other professional specialty (7), supervisors and proprietors, sales occupations (9), farm operators, managers, and supervisors (25) | Executive, administrators, and managers (1), management related occupations (2), engineers, architects and scientists (3), health diagnosing, assessment and treating occupations (4), teachers (5), writers, artists, entertainers, and athletes (6), other professional specialty (7), supervisors and proprietors, sales occupations (9), farm operators, managers, and supervisors (25) |
| White collar, semi-routine | Technicians and related support occupations (8), sales representatives, finance, business, and, commodities except retail (10), secretaries, stenographers, and typists (12), information clerks (13), records processing occupations (14), material recording, scheduling, and distributing clerks (15), miscellaneous administrative support occupations (16), health service occupations (22) | Technicians and related support occupations (8), sales representatives, finance, business, and, commodities except retail (10), secretaries, stenographers, and typists (12), information clerks (13), records processing occupations (14), material recording, scheduling, and distributing clerks (15), miscellaneous administrative support occupations (16), health service occupations (22) |
| Blue collar,  high skill | Vehicle and mobile equipment mechanics and repairers (28), other mechanics and repairers (29), construction trades (30), extractive and precision production occupations (31), armed forces (NHANES III variable HAS17=2) | Vehicle and mobile equipment mechanics and repairers (28), other mechanics and repairers (29), construction trades (30), extractive and precision production occupations (31), armed forces (NHANES 1999-2000 variable OCQ390G=3; NHANES 2001-2002 variable OCD390G=3) |
| Blue collar, semi-routine | Sales workers, retail and personal services (11), private household occupations (17), protective service occupations (18), waiters and waitresses (19), cooks (20), miscellaneous food preparation and service occupations (21), cleaning and building service occupations (23), personal service occupations (24), farm and nursery workers (26), related agricultural, forestry, and fishing occupations (27), textile, apparel, and furnishing machine operators (32), machine operators, assorted materials (33), fabricators, assemblers, inspectors, and samplers (34), motor vehicle operators (35), other transportation and material moving occupations (36), construction laborers (37), laborers, except construction (38), freight, stock, and material movers, hand (39), other handlers, equipment cleaners, and handlers (40) | Sales workers, retail and personal services (11), private household occupations (17), protective service occupations (18), waiters and waitresses (19), cooks (20), miscellaneous food preparation and service occupations (21), cleaning and building service occupations (23), personal service occupations (24), farm and nursery workers (26), related agricultural, forestry, and fishing occupations (27), textile, apparel, and furnishing machine operators (32), machine operators, assorted materials (33), fabricators, assemblers, inspectors, and samplers (34), motor vehicle operators (35), other transportation and material moving occupations (36), construction laborers (37), laborers, except construction (38), freight, stock, and material movers, hand (39), other helpers, equipment cleaners, and hand packages and laborers (40) |
| Never worked | Never worked (NHANES III variable HAS17=1) | Never worked (NHANES 1999-2000 variable OCQ390G=4; NHANES 2001-2002 variable OCD390G=4) |
