# Supplementary material for: Income, inflammation and cancer mortality: a study of U.S. National Health and Nutrition Examination Survey mortality follow-up cohorts
Source: BMC Public Health. 2020 Nov 26;20:1805. doi: 10.1186/s12889-020-09923-8 (PMC7689964; doi:10.1186/s12889-020-09923-8)
Supplement: Supplementary file 2 — Additional file 2: Supplemental Table 2. Demographic, Socioeconomic, and Behavioral Characteristics Associated with CRP Levels. Association analysis between CRP and factors [file 12889_2020_9923_MOESM2_ESM.docx]

Supplemental Table 2. Association of Cancer Mortality and Inflammatory Markers of All Participants

|  | **Model 1-Unadjusted** | | | **Model 2-Demographic Adjusted^a^** | | | **Model 3-Socioeconomic Status Adjusted^b^** | | | **Model 4-Behavioral Factors Adjusted^c^** | | |
| --- | --- | --- | --- | --- | --- | --- | --- | --- | --- | --- | --- | --- |
|  | **HR** | **95% CI** | **P-value** | **HR** | **95% CI** | **P-value** | **HR** | **95% CI** | **P-value** | **HR** | **95% CI** | **P-value** |
| ***NHANES III 1988-1994*** |  |  |  |  |  |  |  |  |  |  |  |  |
| **C-Reactive Protein^d^** |  |  |  |  |  |  |  |  |  |  |  |  |
| <0.22 mg/dL | 1 |  |  | 1 |  |  | 1 |  |  | 1 |  |  |
| 0.22-0.99 mg/dL | 1.11 | 0.87-1.42 | 0.41 | 1.14 | 0.89-1.46 | 0.28 | 1.09 | 0.85-1.39 | 0.51 | 1.01 | 0.81-1.25 | 0.94 |
| >0.99 mg/dL | 1.76 | 1.26-2.46 | 0.001 | 1.87 | 1.35-2.59 | <0.001 | 1.73 | 1.23-2.41 | 0.002 | 1.43 | 0.99-2.08 | 0.06 |
| **Fibrinogen^d^** | 1.000 | 1.001-1.003 | 0.001 | 1.002 | 1.001-1.003 | <0.001 | 1.002 | 1.001-1.002 | 0.001 | 1.001 | 1.00-1.002 | 0.11 |
| ***NHANES 1999-2002*** |  |  |  |  |  |  |  |  |  |  |  |  |
| **C-Reactive Protein^d^** |  |  |  |  |  |  |  |  |  |  |  |  |
| <0.22 mg/dL | 1 |  |  | 1 |  |  | 1 |  |  | 1 |  |  |
| 0.22-0.99 mg/dL | 1.05 | 0.60-1.81 | 0.87 | 1.23 | 0.69-2.20 | 0.47 | 1.06 | 0.60-1.86 | 0.84 | 0.87 | 0.51-1.48 | 0.60 |
| >0.99 mg/dL | 1.95 | 1.16-3.27 | 0.01 | 2.33 | 1.32-4.11 | 0.01 | 1.88 | 1.12-3.15 | 0.02 | 1.44 | 0.87-2.39 | 0.15 |
| **Fibrinogen^d^** | 1.000 | 0.999-1.003 | 0.28 | 1.001 | 0.999-1.004 | 0.18 | 1.001 | 0.999-1.003 | 0.30 | 1.000 | 0.998-1.002 | 0.92 |

^a^ Adjusted for race and gender
^b^ Adjusted for income, education, and occupation
^c^ Adjusted for body mass index, physical activity, and smoking status
^d^ Each of the inflammatory markers were examined in separate multivariate analysis
