# Supplementary material for: Income, inflammation and cancer mortality: a study of U.S. National Health and Nutrition Examination Survey mortality follow-up cohorts
Source: BMC Public Health. 2020 Nov 26;20:1805. doi: 10.1186/s12889-020-09923-8 (PMC7689964; doi:10.1186/s12889-020-09923-8)
Supplement: Supplementary file 3 — Additional file 3: Supplemental Table 3. Demographic, Socioeconomic, and Behavioral Characteristics Associated with Fibrinogen Levels. Association analysis between fibrinogen and factors [file 12889_2020_9923_MOESM3_ESM.docx]

Supplemental Table 3. Demographic, Socioeconomic, and Behavioral Characteristics Associated with CRP Levels

| **NHANES III 1988-1994** | | | | | **NHANES 1999-2002** | | | |
| --- | --- | --- | --- | --- | --- | --- | --- | --- |
| **Characteristics** | **C-Reactive Protein** | | | **P-value** | **C-Reactive Protein** | | | **P-value** |
|  | **<0.22 mg/dL** | **0.22-0.99 mg/dL** | **>0.99 mg/dL** |  | **<0.22 mg/dL** | **0.22-0.99 mg/dL** | **>0.99 mg/dL** |  |
| ***Age (years)*** |  |  |  | <0.001^a^ |  |  |  | <0.001^a^ |
| Younger than 55 yrs | 72.9% | 20.1% | 7.0% |  | 51.4% | 38.8% | 9.8% |  |
| 55 yrs or older | 60.1% | 28.8% | 11.0% |  | 39.3% | 48.9% | 11.8% |  |
| ***Race/Ethnicity*** |  |  |  | <0.001^a^ |  |  |  | 0.002^a^ |
| White | 67.6% | 23.7% | 8.7% |  | 45.1% | 45.0% | 9.9% |  |
| Black | 54.5% | 30.2% | 15.2% |  | 36.2% | 44.9% | 18.8% |  |
| Hispanics/Other | 65.6% | 27.1% | 7.3% |  | 50.2% | 39.6% | 10.3% |  |
| ***Gender*** |  |  |  | <0.001^a^ |  |  |  | <0.001^a^ |
| Male | 72.4% | 21.4% | 6.3% |  | 52.8% | 39.6% | 7.6% |  |
| Female | 60.4% | 27.8% | 11.8% |  | 37.5% | 48.5% | 14.0% |  |
| ***Education*** |  |  |  | <0.001^a^ |  |  |  | <0.001^a^ |
| Below High School | 58.9% | 29.1% | 12.0% |  | 34.6% | 49.0% | 16.4% |  |
| High School/Equivalent | 63.2% | 27.0% | 9.8% |  | 43.7% | 44.2% | 12.1% |  |
| Above High School | 74.1% | 19.5% | 6.4% |  | 50.6% | 42.0% | 7.5% |  |
| ***Occupation*** |  |  |  | <0.001^a^ |  |  |  | 0.001^a^ |
| Not Working | 60.4% | 27.8% | 11.8% |  | 31.4% | 45.0% | 23.6% |  |
| White Collar and Professional | 29.3% | 23.3% | 17.5% |  | 52.9% | 40.5% | 6.6% |  |
| White Collar, Semi-Routine | 22.8% | 22.6% | 22.5% |  | 40.3% | 46.6% | 13.1% |  |
| Blue Collar, High Skill | 66.1% | 26.6% | 7.3% |  | 47.3% | 43.5% | 9.3% |  |
| Blue Collar, Semi-Routine | 61.5% | 26.3% | 12.2% |  | 39.7% | 46.7% | 13.6% |  |
| ***Body Mass Index (kg/m^2^)*** |  |  |  | <0.001^a^ |  |  |  | <0.001^a^ |
| Underweight | 79.2% | 7.3% | 13.5% |  | 72.0% | 24.7% | 3.3% |  |
| Normal | 79.1% | 15.7% | 5.1% |  | 64.8% | 29.1% | 6.1% |  |
| Overweight | 67.8% | 25.0% | 7.2% |  | 47.4% | 43.7% | 8.9% |  |
| Obese | 46.3% | 36.9% | 16.8% |  | 25.0% | 57.7% | 17.2% |  |
| ***Smoking*** |  |  |  | 0.01^a^ |  |  |  | 0.12^a^ |
| Never | 68.2% | 23.8% | 7.9% |  | 46.0% | 43.6% | 10.4% |  |
| Former | 66.6% | 24.4% | 9.0% |  | 47.1% | 43.6% | 9.3% |  |
| Current | 61.6% | 26.8% | 11.5% |  | 39.8% | 46.2% | 14.0% |  |
| ***Physical Activity^b^*** |  |  |  | <.0001^a^ |  |  |  | <0.001^a^ |
| More Active | 73.8% | 20.6% | 5.5% |  | 45.2% | 45.9% | 8.9% |  |
| Less Active | 57.5% | 27.2% | 15.3% |  | 30.7% | 51.4% | 18.0% |  |
| About Same | 63.5% | 27.2% | 9.3% |  | 52.0% | 38.9% | 9.1% |  |

^a^ Chi-squared tests were used for examining the association of inflammatory markers and categorical factors
^b^ Physical activity compared to others of the same age
