# Supplementary material for: Income, inflammation and cancer mortality: a study of U.S. National Health and Nutrition Examination Survey mortality follow-up cohorts
Source: BMC Public Health. 2020 Nov 26;20:1805. doi: 10.1186/s12889-020-09923-8 (PMC7689964; doi:10.1186/s12889-020-09923-8)
Supplement: Supplementary file 4 — Additional file 4: Supplemental Table 4. Association of Cancer Mortality and Inflammatory Markers of All Participants. Cox-proportional hazard analysis of unadjusted (Model 1), demographic adjusted (Model 2), socioeconomic status adjusted (Model 3), and behavioral factors adjusted (Model 4). [file 12889_2020_9923_MOESM4_ESM.docx]

Supplemental Table 4. Demographic, Socioeconomic, and Behavioral Characteristics Associated with Fibrinogen Levels

|  | **NHANES III 1988-1994** | | **NHANES 1999-2002** | |
| --- | --- | --- | --- | --- |
| **Characteristics** | **Mean Fibrinogen (mg/dL)** | **P-value** | **Mean Fibrinogen (mg/dL)** | **P-value** |
| ***Age (years)*** |  | <0.001^a^ |  | <0.001^a^ |
| Younger than 55 yrs | 287 |  | 352 |  |
| 55 yrs or older | 317 |  | 381 |  |
| ***Race/Ethnicity*** |  | <0.001^a^ |  | <0.001^a^ |
| White | 301 |  | 364 |  |
| Black | 318 |  | 393 |  |
| Hispanics/Other | 307 |  | 365 |  |
| ***Gender*** |  | <0.001^a^ |  | 0.001^a^ |
| Male | 295 |  | 359 |  |
| Female | 310 |  | 375 |  |
| ***Education*** |  | <0.001^a^ |  | <0.001^a^ |
| Below High School | 320 |  | 392 |  |
| High School/Equivalent | 303 |  | 367 |  |
| Above High School | 290 |  | 355 |  |
| ***Occupation*** |  | 0.003^a^ |  | <0.001^a^ |
| Not Working | 323 |  | 415 |  |
| White Collar and Professional | 295 |  | 351 |  |
| White Collar, Semi-Routine | 301 |  | 374 |  |
| Blue Collar, High Skill | 304 |  | 365 |  |
| Blue Collar, Semi-Routine | 308 |  | 377 |  |
| ***Body Mass Index (kg/m^2^)*** |  | <0.001^a^ |  | <0.001^a^ |
| Underweight | 294 |  | 345 |  |
| Normal | 293 |  | 344 |  |
| Overweight | 299 |  | 367 |  |
| Obese | 321 |  | 387 |  |
| ***Smoking*** |  | <0.001^a^ |  | <0.001^a^ |
| Never | 298 |  | 364 |  |
| Former | 300 |  | 362 |  |
| Current | 315 |  | 382 |  |
| ***Physical Activity^b^*** |  | <0.001^a^ |  | <0.001^a^ |
| More Active | 302 |  | 360 |  |
| Less Active | 319 |  | 387 |  |
| About Same | 295 |  | 364 |  |

^a^ T-tests were used for examining the association of inflammatory markers and categorical factors
^b^ Physical activity compared to others of the same age
