# Supplementary figures and images for: Income, inflammation and cancer mortality: a study of U.S. National Health and Nutrition Examination Survey mortality follow-up cohorts
Source: BMC Public Health. 2020 Nov 26;20:1805. doi: 10.1186/s12889-020-09923-8 (PMC7689964; doi:10.1186/s12889-020-09923-8)

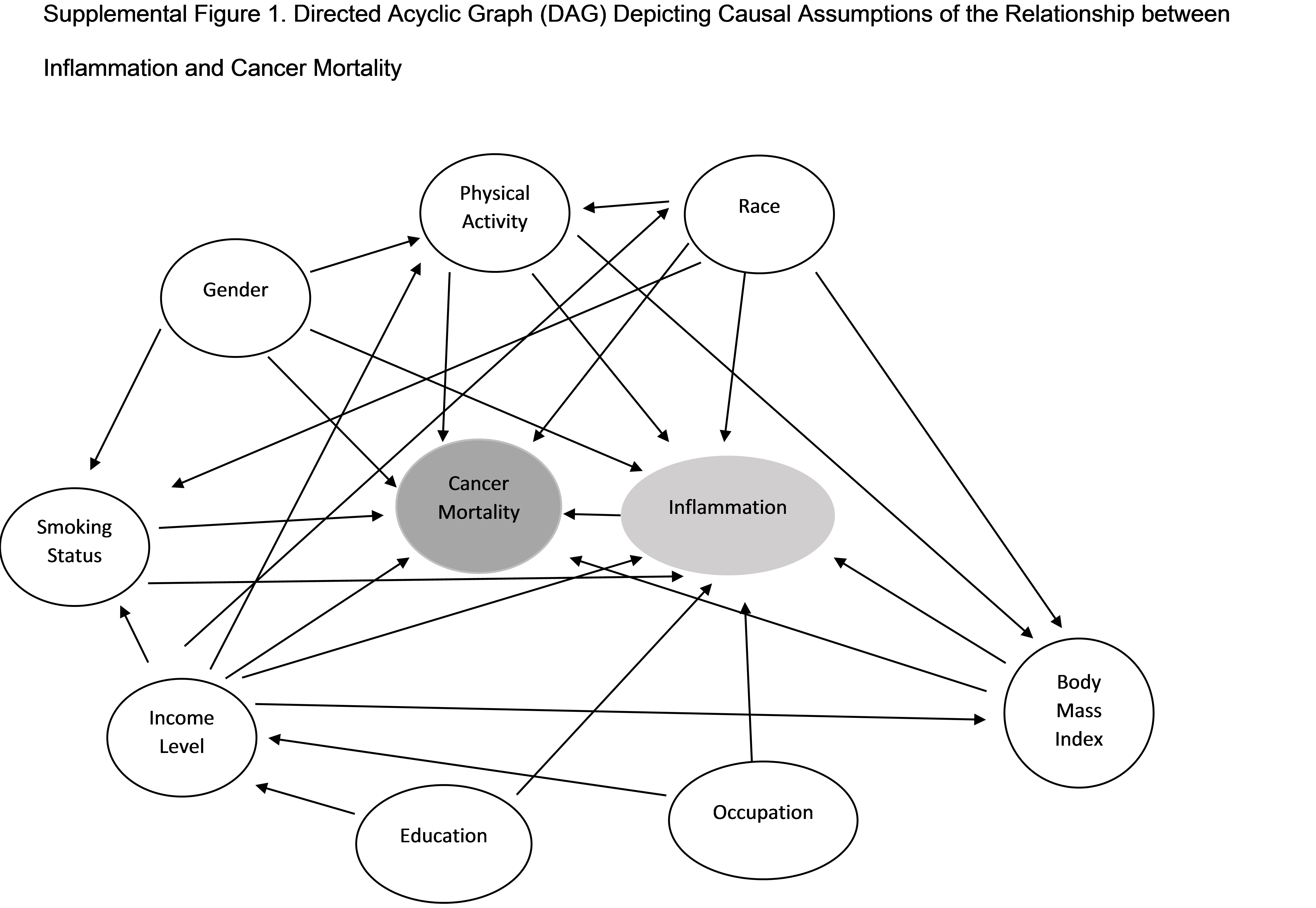

Supplement: Supplementary file 5 — Additional file 5: Supplemental Figure 1. Directed Acyclic Graph (DAG) depicting causal assumptions of inflammation and cancer mortality. Directed Acyclic Graph describes the relationship between inflammation and cancer mortality as well as potential confounders that is associated with both inflammation and cancer mortality. [file 12889_2020_9923_MOESM5_ESM.jpg]

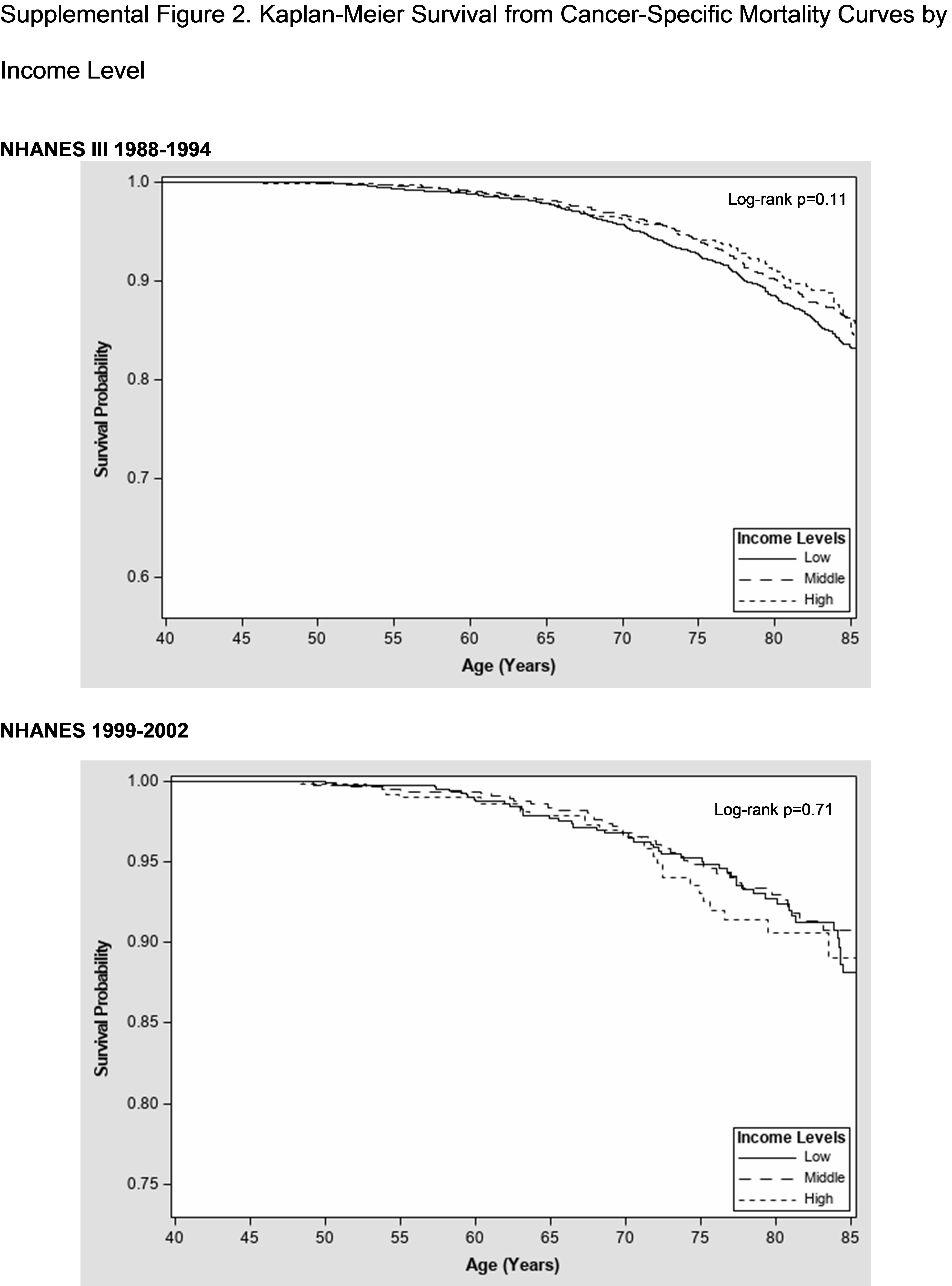

Supplement: Supplementary file 6 — Additional file 6: Supplemental Figure 2. Kaplan-Meier Survival from Cancer-Specific Mortality Curves by Income Level. Differences in survival outcomes stratified by income levels [file 12889_2020_9923_MOESM6_ESM.jpg]

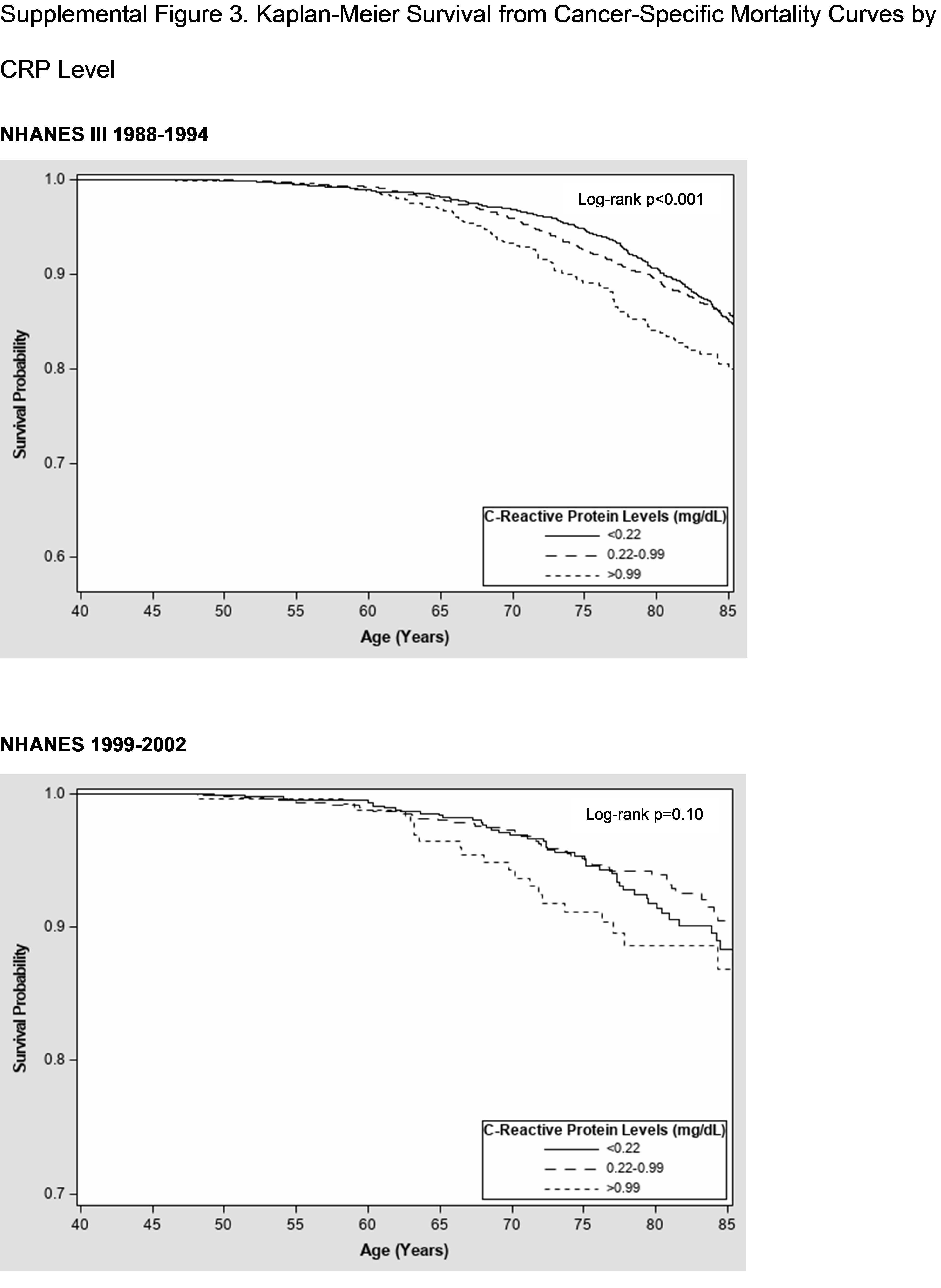

Supplement: Supplementary file 7 — Additional file 7: Supplemental Figure 3. Kaplan-Meier Survival from Cancer-Specific Mortality Curves by CRP Level. Differences in survival outcomes stratified by CRP levels [file 12889_2020_9923_MOESM7_ESM.jpg]

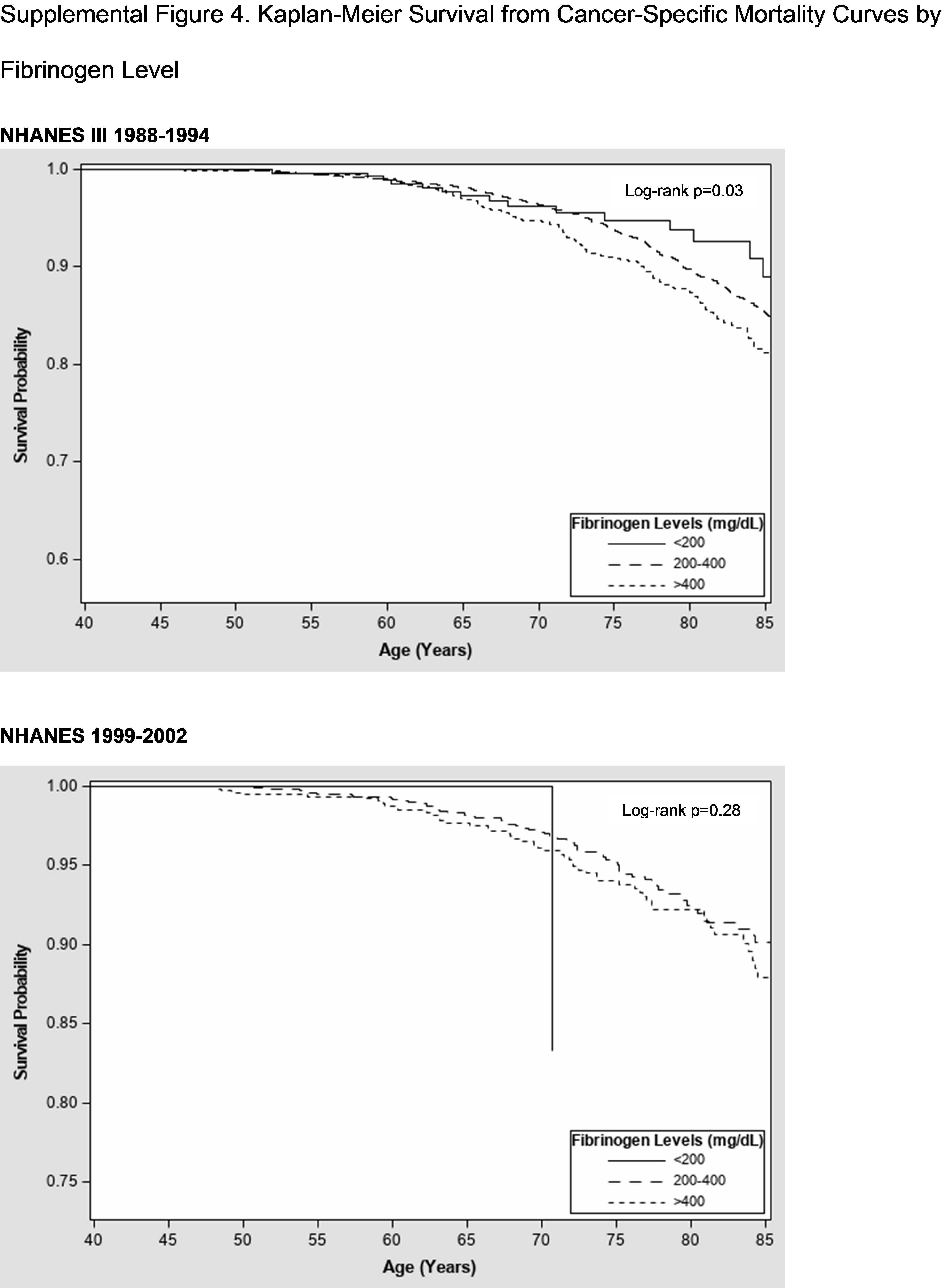

Supplement: Supplementary file 8 — Additional file 8: Supplemental Figure 4. Kaplan-Meier Survival from Cancer-Specific Mortality Curves by Fibrinogen Level. Differences in survival outcomes stratified by fibrinogen levels [file 12889_2020_9923_MOESM8_ESM.jpg]

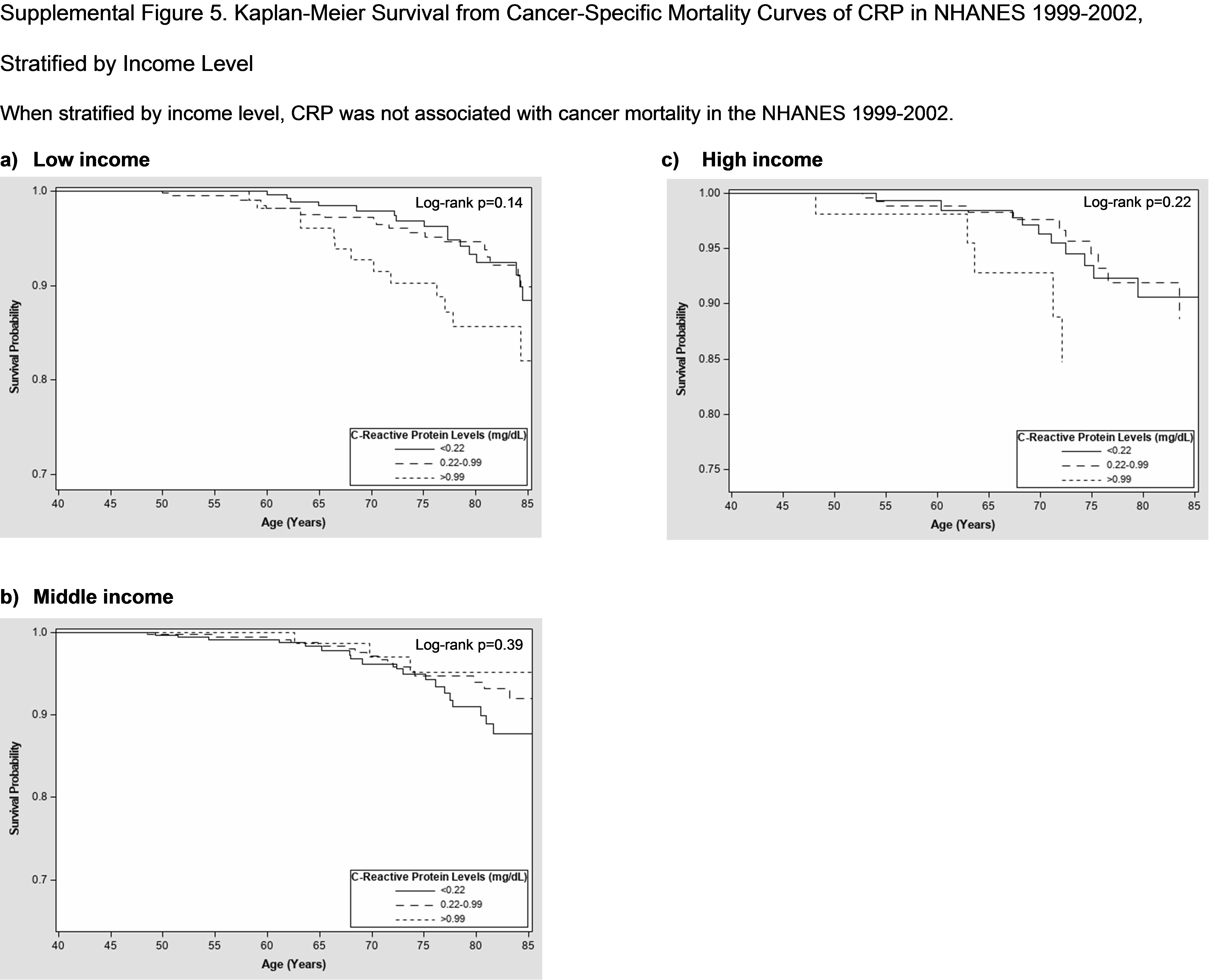

Supplement: Supplementary file 9 — Additional file 9: Supplemental Figure 5. Kaplan-Meier Survival from Cancer-Specific Mortality Curves of CRP in NHANES 1999–2002, Stratified by Income Level. Differences in survival outcomes stratified by CRP and income levels [file 12889_2020_9923_MOESM9_ESM.jpg]
